# Effect of *Schistosoma mansoni* Infection on Innate and HIV-1-Specific T-Cell Immune Responses in HIV-1-Infected Ugandan Fisher Folk

**DOI:** 10.1089/aid.2015.0274

**Published:** 2016-07-01

**Authors:** Andrew Ekii Obuku, Gershim Asiki, Andrew Abaasa, Isaac Ssonko, Alexandre Harari, Govert J. van Dam, Paul L. Corstjens, Moses Joloba, Song Ding, Juliet Mpendo, Leslie Nielsen, Anatoli Kamali, Alison M. Elliott, Giuseppe Pantaleo, Pontiano Kaleebu, Pietro Pala

**Affiliations:** ^1^ Basic Science Programme, Medical Research Council/Uganda Virus Research Institute, Uganda Research Unit on AIDS, Entebbe, Uganda.; ^2^ HIV Prevention and Epidemiology Programme, Medical Research Council/Uganda Virus Research Institute, Uganda Research Unit on AIDS, Entebbe, Uganda.; ^3^ Division of Immunology and Allergy, Department of Medicine, Lausanne University Teaching Hospital, Lausanne, Switzerland.; ^4^ Leiden University Medical Center, Leiden, the Netherlands.; ^5^ Department of Medical Microbiology, Makerere University College of Health Sciences, Kampala, Uganda.; ^6^ EuroVacc Foundation, Amsterdam, the Netherlands.; ^7^ Uganda Virus Research Institute/International AIDS Vaccine Initiative, Entebbe, Uganda.; ^8^ Co-Infections Programme, Medical Research Council/Uganda Virus Research Institute, Uganda Research Unit on AIDS, Entebbe, Uganda.; ^9^ Department of Clinical Research, London School of Hygiene and Tropical Medicine, London, United Kingdom.

## Abstract

In Uganda, fisher folk have HIV prevalence rates, about four times higher than the national average, and are often coinfected with *Schistosoma mansoni*. We hypothesized that innate immune responses and HIV-specific Th1 immune responses might be downmodulated in HIV/*S. mansoni-*coinfected individuals compared with HIV+/*S. mansoni*-negative individuals. We stimulated whole blood with innate receptor agonists and analyzed supernatant cytokines by Luminex. We evaluated HIV-specific responses by intracellular cytokine staining for IFN-γ, IL-2, and TNF-α. We found that the plasma viral load and CD4 count were similar between the HIV+SM+ and HIV+SM− individuals. In addition, the TNF-α response to the imidazoquinoline compound CL097 and β-1, 3-glucan (curdlan), was significantly higher in HIV/*S. mansoni-*coinfected individuals compared with HIV only-infected individuals. The frequency of HIV-specific IFN-γ+IL-2–TNF-α− CD8 T cells and IFN-γ+IL-2–TNF-α+ CD4 T cells was significantly higher in HIV/*S. mansoni-*coinfected individuals compared with HIV only-infected individuals. These findings do not support the hypothesis that *S. mansoni* downmodulates innate or HIV-specific Th1 responses in HIV/*S. mansoni-*coinfected individuals.

## Introduction

In Uganda, the national HIV prevalence fluctuates at about 7%. However, high-risk groups such as the fishing communities on the shores of Lake Victoria have prevalence about three to four times that of the national average.^1^ In addition, the *Schistosoma mansoni* prevalence in the fishing communities living on the shores of Lake Victoria is about 50%. Given the geographic overlap in occurrence, HIV and *S. mansoni* coinfections occur in these fishing communities.^2,3^

Mortality as a result of schistosomiasis is low, with possible subtle effects of schistosomiasis, such as the extent to which schistosomiasis influences the host response to other infections such as HIV, tuberculosis, and malaria, and the response to vaccines.

The HIV (nonenvelope) specific antiviral T-cell immune response is dominated by the secretion of IFN-γ and TNF-α (Th1 profile)^4^ and IL-17 (Th17 profile), whereas *S. mansoni* infection in humans is predominantly characterized by secretion of IL-4, IL-5, and IL-13 (Th2 profile) and IL-17 (Th17 profile) in the acute phase and a regulatory phenotype (T regs) in the chronic phase.^5^ In coinfection of HIV and *S. mansoni*, scanty data are available on the preferential development, differentiation, and expansion of HIV-specific Th1, Th2, or Th17 subpopulations.

The human innate immune system has developed pattern recognition receptors, including nucleotide oligomerization domain (NOD)-like receptors (NLRs), Toll-like receptors (TLRs), and retinoic acid-inducible gene-1 (RIG-I)-like receptors (RLRs), used to recognize pathogen-associated molecular patterns (PAMPs). HIV and its proteins have been observed to have contrasting effects on TLR-related triggering, for example, TLR 2 triggering is associated with increased HIV transfer from dendritic cells (DCs) to CD4 T cells, while TLR 4 triggering is associated with decreased HIV transfer.^6^ HIV and its proteins also trigger TLR 7/8 and TLR 7/9, resulting in the potent activation of DCs and the release of copious amounts of IFN-α, IFN-β, and TNF-α, promoting inflammation and immune activation, and shutting down viral replication.^7,8^ However, gp120 disrupts TLR-9-mediated plasmacytoid DC activation and NK activation, and gp120 treatment suppresses TLR-9-induced secretion of IFN-α and IFN-β.^9^

*S. mansoni*-derived molecules have been associated with induction of cytokines with divergent influences on the T-cell response profile at priming. In animal models, a Th1-type response can be seen in the initial 4–5 weeks after infection, switching to Th2 when egg production starts.^10^ Secretion of IL-12p40 and IL-10 by thioglycollate-induced murine macrophages stimulated with *S. mansoni* antigen is largely dependent upon the presence of a functional TLR 4.^11^ However, dextran-conjugated lacto-*N*-fucopentaose III, containing the characteristic sugar moiety present in soluble egg antigen (SEA), ligates TLR 4 and DC SIGN^12^ and skews immature DCs to DC2, facilitating secretion of higher quantities of IL-4 and less IFN-α.^13,14^ Glycolipids from *S. mansoni* worms signal through TLR 4, causing DC maturation with an inflammatory bias and a Th1 profile in cocultured naïve T cells.^15^ Lipids from schistosome eggs are TLR 2 ligands and drive DCs to produce IL-10.^16^ The combined ligation of TLR2 and TLR4 by different bacterial and helminth molecules induces distinct gene expression signatures correlated with Th1 or Th2 polarization in monocyte-derived human DCs.^17^ SEA has also been shown to induce a Th2 bias in mice lacking TLR2, TLR4, or MyD88.^18^ DC SIGN, a receptor on DCs, is ligated by both gp120 and SEA, constituting an opportunity for competitive interference.^12^ Both HIV and *S. mansoni* utilize TLRs in their mechanism of establishing infection. This cross utilization could affect the ability of cells from *S. mansoni*-infected individuals to mount an immune response to an incoming pathogen such as HIV, and the triggering of TLR 2 or 4 by *S. mansoni*, cercariae, egg, or worm antigen could affect HIV transfer from DCs to CD4 T cells.

To investigate the immunological interaction between HIV and *S. mansoni*, we have characterized cellular innate responses to TLR agonists, and the adaptive immune response to HIV antigen, in HIV+/*S. mansoni*-negative individuals and HIV+ individuals coinfected with *S. mansoni*. We report that (a) TNF-α secretion in response to CL097 and curdlan stimulation is significantly higher in HIV/*S. mansoni-*coinfected individuals compared with HIV+/*S. mansoni*-negative individuals and (b) the frequency of HIV-specific IFN-γ+IL-2–TNF-α– CD8 T cells and IFN-γ+IL-2–TNF-α+ CD4 T cells was significantly higher in HIV/*S. mansoni-*coinfected individuals compared with HIV only-infected individuals.

These findings do not support the hypothesis that *S. mansoni* downmodulates innate or HIV-specific Th1 responses in HIV/*S. mansoni-*coinfected individuals.

## Materials and Methods

### Ethics statement

The Institutional Ethics Review Board of the Uganda Virus Research Institute and the Uganda National Council of Science and Technology approved the study. All subjects provided written informed consent.

### Study groups

Fifty HIV-infected individuals were recalled 22–537 estimated days post-HIV infection and after exiting a larger 18-month-long study investigating the HIV incidence and risk factors for HIV acquisition in the Lambu, Kamuwunga, Nakiwogo, Kasenyi, and Nsazi fish landing sites on the shores and islands of Lake Victoria.^3,19^

*S. mansoni* infection was determined by the standard Kato–Katz method on three consecutive stool samples at recall visits. *S. mansoni* infection status before HIV acquisition was determined by circulating anodic antigen (CAA) assay^20^ in sera samples collected at months 0, 6, 12, and 18 during the previous HIV incidence study. The HIV seroconversion date was estimated as the mid-date between the date of last seronegative test and the date of the first seropositive test.

### Whole blood assay

Heparinized blood was diluted in a 1:1 ratio with Roswell Park Memorial Institute (RPMI) 1640+HEPES (Sigma). One hundred microliters of diluted blood and 100 μl of each stimulus were added to a round-bottomed 96-well plate and incubated at 5% CO_2_ 37°C for 24 h. The stimuli used were lipopolysaccharide [LPS (100 ng/ml), InvivoGen]; PAM3CSKKKK [PAM (100 ng/ml), EMC Microcollections, Tübingen, Germany]; synthetic lipoprotein, mycoplasma type [FSL-1 (50 ng/ml), InvivoGen]; imidazoquinoline compound [CL097 (1 μg/ml), InvivoGen]; CpG-ODN2006 [CpG (5 μg/ml), InvivoGen]; curdlan [Cur (100 μg/ml); Wako Chemicals]; and phytohemagglutinin [PHA (10 μg/ml), Sigma]. An equivalent volume of culture medium was used as negative control. Two 75-μl aliquots of culture supernatants were harvested and frozen.

A custom-made Bio-Rad Luminex kit (Cat. No. L5007NCLYV) was used to measure IL-12p70, TNF-α, IFN-γ, IL-10, and IL-13 in culture supernatants following the manufacturer's instructions with modifications for testing using half volumes. Briefly, 50 μl of thawed culture supernatant was added to each well of a flat-bottomed plate containing 0.5× coupled beads and incubated on a shaker at room temperature (RT) for 30′. After washing, 0.5× of detection antibodies was added and incubated at RT for further 30′. The beads were washed, then 0.5× of streptavidin-PE was added and incubated at RT for 10′ and washed. Eighty microliters of buffer was added and detection was done using a Bio-Plex 200 system with Bio-Plex manager, version 6.0. Cytokine concentrations were determined using standard curves and limits of quantitation calculated by the software according to a four- or five-parameter logistic model. Since the upper limit of quantitation varied between different assays, results above the upper limit of quantitation were excluded. Background responses (cultures with RPMI1640/HEPES) were subtracted from the corresponding stimulated responses and any negative values were set to zero.

### Intracellular cytokine staining assay

Cryopreserved peripheral blood mononuclear cells (PBMC) (1–2 × 10^6^) were thawed and rested for 6 h and stimulated overnight in 1 ml of complete medium [RPMI (Sigma), 10% fetal bovine serum (PAA), 100 μg/ml penicillin, 100 units/ml streptomycin (Lonza)] in the presence of Golgiplug (1 μl/ml, BD), anti-CD28 (0.5 μg/ml, BD), and 1 μg/ml of Gag potential T-cell epitope (PTE) pools 1 and 2 peptides^21^. Staphylococcus enterotoxin B (Sigma) stimulation (200 ng/ml) was used as a positive control. Medium alone was used as a negative control. At the end of the stimulation period, cells were stained for 20′ at 4°C using Aqua LIVE/DEAD cell stain (Invitrogen), fixed, and permeabilized (Cytofix/Cytoperm; BD) at RT for 20′, and then stained at RT for 20′ with CD3-APC H7, CD4-PE-CF594, CD8-Pacific Blue, TNF-α-FITC, IFN-γ-APC, and IL-2-PE antibodies (all from BD). Cellfix (BD) was added and 0.6–1 × 10^6^ lymphocyte-gated events were acquired on an LSRII SORP (BD). FlowJo version 9.7.4 (Treestar) was used in the analysis. To exclude doublets, data on FSC-A and FSC-H were used. Aqua-positive cells were also excluded (Supplementary Fig. S1; Supplementary Data are available online at www.liebertpub.com/aid).

Only individuals classified as responders to each peptide pool were compared. A peptide pool was considered to stimulate a cytokine response if any of the IFN-γ OR IL-2 OR TNF-α responses were ≥0.05% after background subtraction.

For the polyfunctionality analysis, boolean-gated unstimulated responses were subtracted from corresponding responses to stimuli and any resulting values below 0.01% were set to 0%.

### Statistical analyses

Analyses were done using GraphPad Prism 6.0. Student's *t*-test was used to compare means between the two groups. The Holm–Šídák method was used to correct for multiple comparisons.

## Results

### Biomedical characteristics of study participants

Among the 34 participants whose innate immune response was analyzed, 18 were HIV/*S. mansoni* coinfected. The mean age of the HIV+SM+ individuals was 29.3 years and that of HIV+SM− individuals was 29.4 years. The mean plasma viral load among the HIV+SM+ coinfected individuals was 118,058 RNA copies per ml compared with 73,980 RNA copies per ml for the HIV+SM− individuals. The mean CD4 count per microliter of blood among the HIV+SM+ individuals was 716 compared with 651 among the HIV+SM− individuals (Table 1).

**Table 1.** Biomedical Characteristics of Participants in the Innate Response Analysis

	*SM*+		*SM−*	
	*Female*	*Male*	*Female*	*Male*
No. of individuals (*n*)	6	12	9	7
Mean age in years (range)	30 (21–39)	29 (23–41)	29 (20–45)	30 (24–37)
Mean CD4 count per μl of blood (range)	559 (361–779)	795 (362–1,228)	514 (424–623)	826 (431–2,000)
Mean viral load copies per ml (range)	73,294 (2,408–325,713)	140,440 (2,664–786,051)	94,735 (248–338,494)	47,294 (20–104,879)

Among the 38 participants whose adaptive immune response was analyzed, 18 were HIV/*S. mansoni* coinfected. The mean age of the HIV+SM+ individuals was 28.8 years and that of HIV+SM− individuals was 28.4 years. The mean plasma viral load among the HIV+SM+ coinfected individuals was 115,989 RNA copies per ml compared with 123,058 RNA copies per ml for the HIV+SM− individuals. The mean CD4 count per microliter of blood among the HIV+SM+ individuals was 748 compared with 734 among the HIV+SM− individuals (Table 2). Participants were ART-naïve and had not received anthelmintic treatment in the previous 3 months.

**Table 2.** Biomedical Characteristics of Participants in the Adaptive Response Analysis

	*SM*+		*SM−*	
	*Female*	*Male*	*Female*	*Male*
No. of individuals (*n*)	3	15	11	9
Mean age in years (range)	28 (21–39)	29 (23–41)	27 (19–45)	30 (24–37)
Mean CD4 count per μl of blood (range)	580 (361–779)	782 (362–1,228)	701 (436–1,115)	775 (417–2,000)
Mean viral load copies per ml (range)	4,349 (2,408–5,957)	138,317 (2,664–786,051)	185,047 (248–830,432)	47,294 (20–104,879)

### Innate responses: association of *S. mansoni* coinfection with higher TNF-α concentrations in whole blood cultures from HIV+ individuals

We investigated innate cytokine responses in the context of HIV/*S. mansoni* coinfections. Using CAA assays on plasma collected at six monthly intervals during the HIV incidence study, we could ascertain that *S. mansoni* infection preceded the HIV infection in all coinfected cases; therefore, HIV would not have influenced the early course of *S. mansoni* infection. Samples from 34 HIV-infected individuals, 18 of whom were coinfected with *S. mansoni*, were evaluated in these assays. We assayed culture supernatants from 34 individuals instead of 50 individuals because of experimental failures and missing or used up samples.

**FIG. 1.**
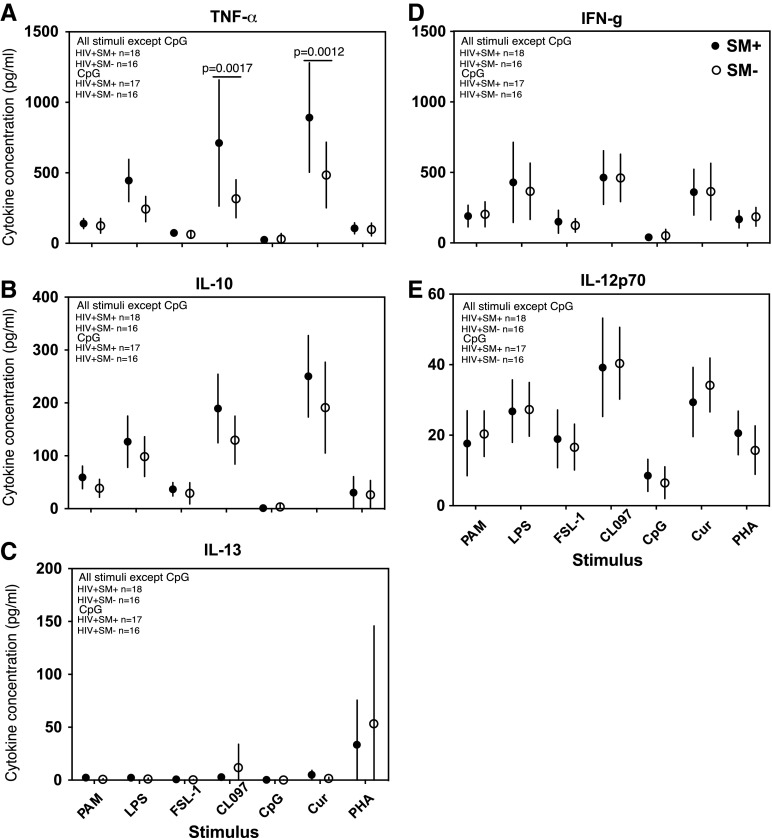
Cytokine response after agonist stimulation. TNF-α **(A)**, IL-10 **(B)**, IL-13 **(C)**, IFN-γ **(D)**, and IL-12p70 **(E)** concentrations (pg/ml) after 24-h whole blood stimulation with PAM, LPS, FSL-1, CL097, CpG, curdlan, and PHA. Only significant *p*-values are indicated in the figure. HIV+SM+ *n* = 18 and HIV+SM− *n* = 16 for all stimuli except CpG where *n* = 17 for the HIV+SM+. Student's *t*-test and the Holm–Šídák correction for multiple comparisons were used to compare the response to each stimuli between HIV/*S. mansoni* coinfected (SM+) and HIV+SM− infected only. Only significant *p*-values are shown.

The mean concentration of TNF-α in whole blood culture supernatants following CL097 and curdlan stimulation of whole blood cultures from HIV+SM+ individuals was significantly higher compared with that of HIV+SM− individuals [711 pg/ml vs. 316 pg/ml, *p* = .0017; 892 pg/ml vs. 484 pg/ml, *p* = .0012, respectively (Fig. 1A)]. In addition, LPS stimulation was associated with somewhat stronger TNF-α responses in HIV+SM+ individuals compared with HIV+SM− individuals (Fig. 1A).

The remaining cytokine responses did not differ significantly between HIV+SM+ and HIV+SM− (Fig. 1B–E).

The innate immune response was not stratified by sex because a nonsignificant difference was observed when the number of males to females was compared (data not shown).

### Adaptive responses to HIV: association of *S. mansoni* coinfection with higher frequency of Gag-specific CD8 T cells producing IFN-γ in HIV+ individuals

**FIG. 2.**
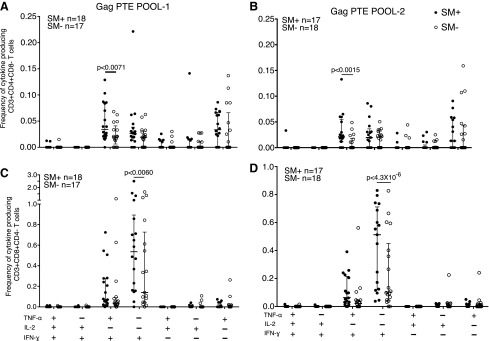
Functional profile of HIV-specific CD4 and CD8 T-cell responses in HIV/*S. mansoni* coinfected and HIV only infection. Analysis of the functional profile of secretion of IFN-γ, IL-2, and TNF-α in response to Gag PTE pool-1 **(A), (C)**, SM + *n* = 18, SM− *n* = 17, and Gag PTE pool 2 **(B), (D)** SM + *n* = 17, SM− *n* = 18. All possible combinations of positive responses are shown on the *x*-axis, whereas the frequency of cytokine-producing CD4 or CD8 T cells is shown on the *y*-axis. Student's *t*-test and the Holm–Šídák correction for multiple comparisons were used to compare the response to each PTE pool between HIV/*S. mansoni-*coinfected (SM+) and HIV+SM− -infected individuals. Only significant *p* values are shown. PTE, potential T-cell epitope.

PBMCs obtained at the same visit as the whole blood for innate responses were stimulated with Gag PTE pools 1 and 2 and we compared IFN-γ, IL-2, and TNF-α production by CD4 and CD8 T cells between HIV+SM+ and HIV+SM− individuals. The mean frequency of CD8 T cells with an IFN-γ+ IL-2− TNF-α– profile in response to Gag PTE pool 1 (0.7% vs. 0.40%, *p* < .0060) and Gag PTE pool 2 (0.43% vs. 0.25%, *p* < 4.3 × 10^−6^) was significantly higher in HIV+SM+ individuals compared with HIV+SM− individuals (Fig. 2C, D).

The mean frequency of CD4 T cells with an IFN-γ+ IL-2− TNF-α+ profile in response to Gag PTE pool 1 (mean 0.05% vs. 0.02%, *p* < .0071) and Gag PTE pool 2 (mean 0.03% vs. 0.01%, *p* < .0015) was also significantly higher in HIV+SM+ individuals compared with HIV+SM− individuals (Fig. 2A, B).

The dominant cytokine response combinations in the CD8 subset were IFN-γ+ IL-2− TNF-α− and IFN-γ+ IL-2− TNF-α+ (Fig. 2C, D) and IFN-γ+ IL-2− TNF-α+ for the CD4 subset (Fig. 2A, B). The CD4 subset had a more even distribution of cells producing 1 or 2 cytokine combinations in both HIV+SM+ and HIV+SM−, but their frequency was lower than among CD8 T cells (Fig. 2A, B). Few cells were IFN-γ+ IL-2+ TNF-α+.

Since we had a significant excess of males in the *S. mansoni-*coinfected participants, we stratified the analysis by sex. The mean frequency of CD4 T cells expressing IFN-γ+ IL-2− TNF-α+ was higher among the HIV+SM+ males compared with HIV+SM− males (mean = 0.035% vs. 0.0033%; *p* = .0005) for Gag PTE pool 2. The frequency of CD8 T cells expressing IFN-γ+ IL-2− TNF-α− was higher among the HIV+SM+ males compared with HIV+SM− males (mean = 0.43% vs. 0.15%; *p* = 4.2 × 10^−8^) for cells stimulated with Gag PTE pool 2.

No statistical significant difference was observed between the HIV+SM+ and HIV+SM− females (data not shown).

## Discussion

We investigated the innate and adaptive cytokine and chemokine profiles in the context of HIV/*S. mansoni* coinfection, where *S. mansoni* infection was acquired before HIV infection.

### Effects on innate immunity

Innate immune cells from HIV+SM+ individuals had a higher secretion of TNF-α in response to CL097 and curdlan stimulations compared with HIV+SM− individuals. We did not observe any significant downregulation of cytokine innate responses in HIV+SM+ individuals compared with HIV+SM− individuals.

TNF-α is a proinflammatory cytokine, which has been connected with the hepatic granuloma response to *S. mansoni* eggs. However, we did not find significant differences in *ex vivo* cytokine production in unstimulated whole blood from HIV+SM+ and HIV+SM− individuals. Instead, we found enhanced responsiveness to imidazoquinoline compound CL097 and curdlan in HIV+SM+ individuals compared with HIV+SM− individuals, suggesting a change in the functional profile of antigen presentation, which could result in increased T-cell responses to antigens presented by TLR7/8- and dectin-expressing APCs that encountered the corresponding TLR ligand *in vivo*. This view is supported by the report that APCs treated with imidazoquinoline ligands of TLR7 and TLR9 or CpG can enhance TNF-α and IFN-γ T-cell responses to HIV peptides *in vitro*^22^ and by our own findings of a bias toward IFN-γ and TNF-α responses in *ex vivo* CD8 T cells specific for HIV Gag.

Stimulation with particulate β-1,3-glucan (curdlan) induced the most enhanced differences in TNF-α response between HIV+SM+ and HIV+SM− individuals. This suggests that *S. mansoni* might be interfering with the β-glucan->dectin-1->Raf-1 signaling pathway, which produces extensive epigenetic reprogramming in the differentiation of monocytes to macrophages^23^ and trained immunity, a form of innate memory.^24^

The identity of the *S. mansoni* products that might be involved is unclear. Blocking experiments with low-molecular-weight soluble glucan laminarin suggest that dectin-1 is not involved in SEA binding^25^; however, the requirement for a particle to activate dectin-1 responses^26^ might be fulfilled by ligands immobilized on the schistosome egg surface.

The presence of the proinflammatory cytokine, TNF-α, is likely sensed directly by the worms that encode a TNF-α receptor,^27^ and egg production is delayed in T-cell-deficient SCID mice, which, however, retain innate sources of TNF-α that could compensate the missing output from T cells.^28^

IFN-γ was associated with protection against liver fibrosis in human infection with *S. mansoni*,^29^ and both IFN-γ and T-bet have been identified as factors restraining severe granuloma pathology associated with IL-17 responses in mouse models of *S. mansoni* infection.^30,31^ Although we could not discern differences between HIV+SM+ versus HIV+SM− individuals in the IFN-γ response to innate stimuli, the frequency of IFN-γ+ CD8 T cells and IFN-γ+TNF-α+ CD4 T cells in response to HIV was significantly enhanced in coinfected individuals.

IL-10 has been suggested as a mechanism through which *S. mansoni* induces downmodulation of Th1 immune responses.^32,33^ In this study, we observed a nonsignificant trend toward higher IL-10 production in whole blood cultures from HIV+SM+ versus HIV+SM−; however, no significant downmodulation of IFN-γ or TNF-α or IL-2 production by either CD4 or CD8 T cell was observed in HIV+SM+ versus HIV+SM− individuals.

### Effects on adaptive T-cell responses

CD4 T cells play a major role in the pathogenesis of HIV and *S. mansoni*. HIV infects and replicates in CD4 T cells, resulting in death of the infected cells and immunodeficiency, whereas in schistosomiasis, CD4 T cells play a role in host resistance to infection^34^ and excretion of parasite eggs.^35^ In this study, we report that the frequency of HIV-specific IFN-γ+ IL-2− TNF-α+ CD4 T cells and IFN-γ+ IL-2− TNF-α− CD8 T cells in response to Gag PTE pools 1 and 2 was significantly higher in the HIV+SM+ individuals compared with HIV+SM− individuals, suggesting that *S. mansoni* infection enhanced HIV-specific Th1 and CD8 T-cell responses. Among HIV+SM+ males only, the frequency of IFN-γ+IL-2-TNF-α+ CD4 T cells and IFN-γ+IL-2− TNF-α− CD8 T cells in response to Gag PTE pool 2 was significantly enhanced in the HIV+SM+ individuals compared with HIV+SM− individuals.

In the context of *S. mansoni* infection, the higher frequency of males with *S. mansoni* infection compared with females is likely due to exposure while fishing, which is largely done by males in the community studied. In the analysis stratified by sex, we did not retain statistically significant differences in acquired responses to HIV in the females. This could be due to biological differences related to sex or could simply reflect the small number of females with *S. mansoni* infection (3 SM+ vs. 10 SM−).

In a coinfection with HIV and *S. mansoni*, pathogens characterized by different cytokine secretion profiles, it is not clear whether the interaction is more detrimental or beneficial for the host when compared with each infection on its own. Chronic inflammation measured by the presence of cytokines such as TNF-α, IFN-γ, and IL-1β has been shown to exacerbate HIV pathogenesis,^36^ and *S. mansoni* might increase HIV disease progression through enhanced immune activation.

Unlike pathologies such as multiple sclerosis where *S. mansoni* SEA seems to downregulate autoimmune inflammation mediated through IFN-γ, TNF-α, and IL-17 by inducing suppressor of cytokine signaling 3 (SOCS3)^37,38^ or coinfection with HTLV-I, where the Th1 type response to the virus is curbed,^39^ the overall profile that emerges from the innate and acquired responses in HIV/*S. mansoni-*coinfected individuals is more proinflammatory than in single HIV infection, although some regulatory effect might be exerted by IL-10, which showed a trend for increased responsiveness to various TLR ligands together with TNF-α and IFN-γ. Since individuals in the present study were infected with HIV for an average of 405 days (105–625 days), we cannot exclude the possibility that downmodulatory effects of *S. mansoni* on HIV immune responses might become detectable over a longer time.

Previous studies have suggested a detrimental effect of *S. mansoni* coinfection on the disease progression in HIV-infected individuals; however, there is no consensus on the benefits of anthelmintic treatment in coinfections with HIV.^40,41^ Praziquantel treatment was followed by changes in the cytokine response to *S. mansoni* toward a Th2 profile, but less so in HIV-coinfected individuals,^42^ and toward a more proinflammatory phenotype in single infections with *S. haematobium.*^43^ We are currently conducting a larger study to discern the effect of annual versus three-monthly praziquantel treatment in HIV+SM+ -coinfected individuals on HIV viral load. Interestingly, *S. mansoni* does not appear to have an effect on HIV acquisition in Ugandan fisher folk,^2,3^ unlike in Tanzanian women.^44^

These conflicting reports reflect the complexity of concurrent responses to multiple antigens and the probable active role of live worms in immune regulation. These results suggest that any detrimental effect of coinfection is more likely due to enhanced HIV disease progression facilitated by an inflammatory environment than to downregulation of anti-HIV responses.

## Supplementary Material

Supplemental data
