# Supplementary material for: Effect of Schistosoma mansoni Infection on Innate and HIV-1-Specific T-Cell Immune Responses in HIV-1-Infected Ugandan Fisher Folk
Source: AIDS Res Hum Retroviruses. 2016 Jul 1;32(7):668–75. doi: 10.1089/aid.2015.0274 (PMC4931742; doi:10.1089/aid.2015.0274)
Supplement: Supplemental data [file Supp_Figure1.pdf]

Supplementary Data

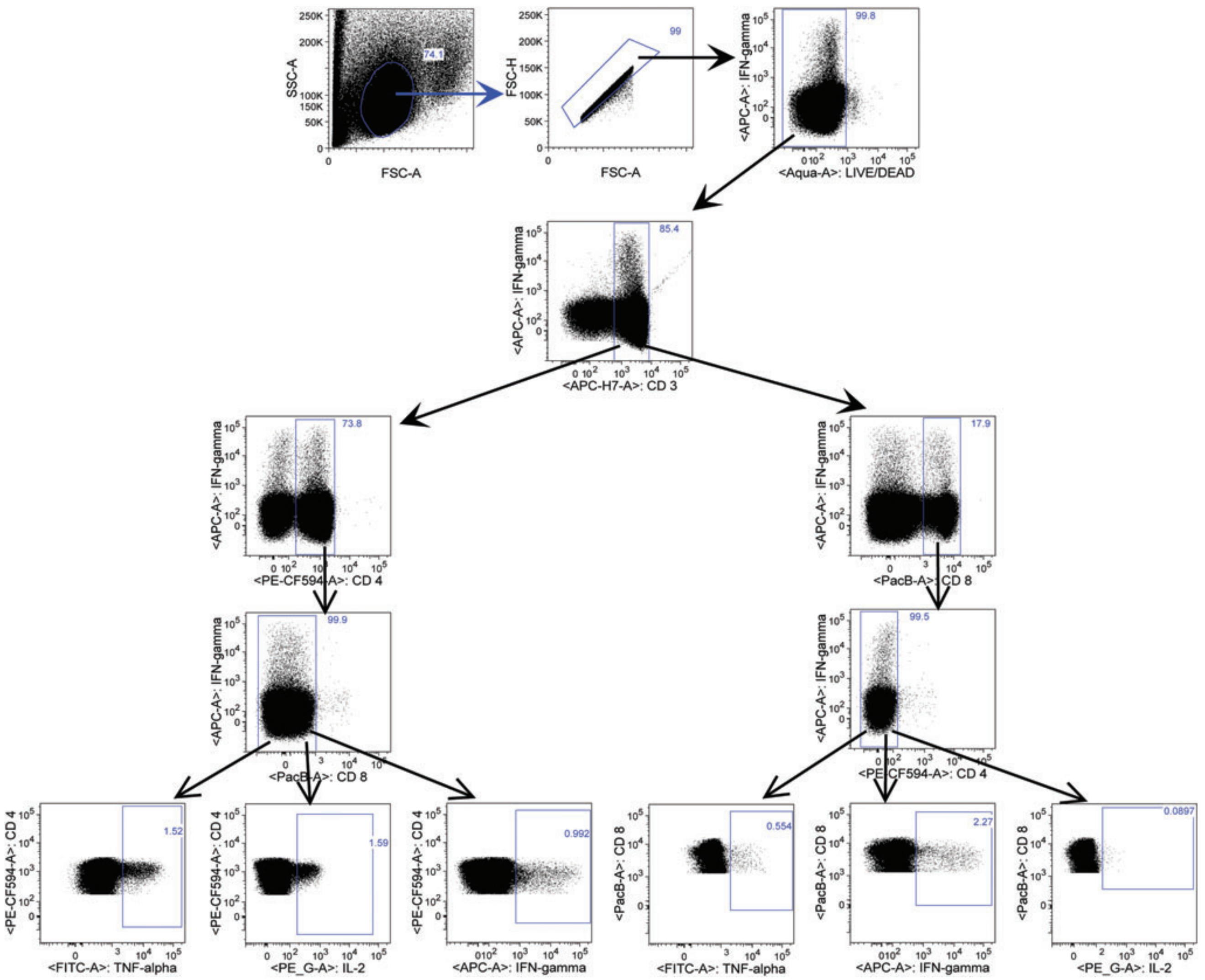

SUPPLEMENTARY FIG. S1. Gating strategy on the adaptive response. Responses shown are from PBMCs stimulated with SEB. PBMC, peripheral blood mononuclear cells; SEB, staphylococcus enterotoxin B.
